# Essential Oil Content of the Rhizome of *Curcuma purpurascens* Bl. (*Temu Tis*) and Its Antiproliferative Effect on Selected Human Carcinoma Cell Lines

**DOI:** 10.1155/2014/397430

**Published:** 2014-08-11

**Authors:** Sok-Lai Hong, Guan-Serm Lee, Syarifah Nur Syed Abdul Rahman, Omer Abdalla Ahmed Hamdi, Khalijah Awang, Nurfina Aznam Nugroho, Sri Nurestri Abd Malek

**Affiliations:** ^1^Institute of Biological Sciences, Faculty of Science, University of Malaya, 50603 Kuala Lumpur, Malaysia; ^2^Department of Chemistry, Faculty of Science, University of Malaya, 50603 Kuala Lumpur, Malaysia; ^3^Faculty of Mathematics and Sciences, Yogyakarta State University, Yogyakarta 55281, Indonesia

## Abstract

*Curcuma purpurascens* Bl., belonging to the Zingiberaceae family, is known as *temu tis* in Yogyakarta, Indonesia. In this study, the hydrodistilled dried ground rhizome oil was investigated for its chemical content and antiproliferative activity against selected human carcinoma cell lines (MCF7, Ca Ski, A549, HT29, and HCT116) and a normal human lung fibroblast cell line (MRC5). Results from GC-MS and GC-FID analysis of the rhizome oil of *temu tis* showed turmerone as the major component, followed by germacrone, *ar*-turmerone, germacrene-B, and curlone. The rhizome oil of *temu tis* exhibited strong cytotoxicity against HT29 cells (IC_50_ value of 4.9 ± 0.4 *μ*g/mL), weak cytotoxicity against A549, Ca Ski, and HCT116 cells (with IC_50_ values of 46.3 ± 0.7, 32.5 ± 1.1, and 35.0 ± 0.3 *μ*g/mL, resp.), and no inhibitory effect against MCF7 cells. It exhibited mild cytotoxicity against a noncancerous human lung fibroblast cell line (MRC5), with an IC_50_ value of 25.2 ± 2.7 *μ*g/mL. This is the first report on the chemical composition of this rhizome's oil and its selective antiproliferative effect on HT29. The obtained data provided a basis for further investigation of the mode of cell death.

## 1. Introduction

*Curcuma* is a renowned genus in the family of Zingiberaceae due to the popularity of turmeric,* Curcuma longa* L. [1].* Curcuma* species are native to several countries of Southeast Asia and extensively cultivated in Bangladesh, India, China, Taiwan, Sri Lanka, Indonesia, Peru, Australia, and the West Indies [2]. The name* Curcuma* was established by Linnaeus (1753), and its generic epithet is derived from the Arabic word “*kurkum*,” meaning yellow colour, which refers to the colour of the rhizomes [3, 4].* Curcuma* has been circumscribed by its cone-like inflorescence of few-flowered, congested bracts and versatile, usually spurred anthers [5].* Curcuma* is mostly grown for its foliage or rhizomes, which are widely used as a starch source, food seasoning or flavours in native dishes, coloured pigments (orange, yellow, citron, amber, blue, greenish-blue, and violet-blue), and ingredients in traditional medicines to treat various ailments, including aches, pains, wounds, liver disorders, and cancers [4, 6, 7].

*Curcuma purpurascens* Bl. is one of the less known* Curcuma* species and is considered of minor importance [4].* C. purpurascens*, locally known as* temu tis* in Yogyakarta, Indonesia, is also known as Solo's (east of Yogyakarta)* temu glenyeh* or* temu blenyeh*, whose scientific name is* Curcuma soloensis* Val. [8]. Villagers from the Kediri district of East Java plant* C. purpurascens* or* temu tis* at the base of Mount Wilis. The rhizomes are dried and ground before being sold to wholesalers as alternative medicine. The powdered rhizomes are usually taken together with other herbs to treat ailments, such as cough and skin infections [8, 9].* Temu tis* can grow up to 1.75 m in height and usually flowers from October to February [9]. Morphologically, the rhizomes of* temu tis* are similar to those of common turmeric (*Curcuma longa*). However, cross-sections of the rhizomes of* temu tis* are slightly bigger and paler in colour in comparison to common turmeric [8]. Hence, the dried ground rhizomes of* temu tis* are often used to adulterate dried ground rhizomes of common turmeric and* Curcuma xanthorrhiza* (locally known as* temulawak*) for higher profit margins [8].

Until now, the phytochemical and biological investigations reported on this plant have been very limited. The plantlet of* temu tis* was reported to be successfully transplanted to soil via propagation using* in vitro* tissue culture methods [9]. The essential oil content of the dried ground rhizomes of* temu tis* showed the presence of only four compounds, namely, turmerone and* ar*-turmerone (as the major components), as well as xanthorrhizol and isofuranogermacrene (also known as curzerene) [8]. Hexane and chloroform extracts of the rhizomes of* C. purpurascens* were reported to exhibit the strongest inhibition against* Candida albicans* [10]. Fractions C and G obtained from the hexane extract and fraction *a* obtained from the chloroform extract were found to have good antifungal activity, comparable to that of the reference standard, miconazole [10]. 1-*α*-Terpineol, *β*-eudesmol, farnesol, 1,6-dimethyl-9-(1-methylethylidene)-5,12-dioxatricyclo[9.1.0.0(4,6)]dodecan-8-one, and caryophyllene oxide were identified in these fractions via gas chromatography-mass spectrometry (GC-MS) [10].

Although* temu tis* is considered one of the less important* Curcuma* species, there is still a need to investigate the chemical constituents of its rhizome oil and its biological activity. Results from chemical investigation of the rhizome oil of* temu tis* can be utilised to authenticate* temu tis* and to determine the presence of adulteration in ground turmeric. Results from biological investigations on the rhizome oil or extracts of* temu tis* would provide more insight into the potential use of this plant for therapeutic purposes. Thus, in the present study, the essential oil obtained from hydrodistilled dried ground rhizome oil of* temu tis* was analysed for its chemical composition, and its antiproliferative activity against selected human carcinoma cell lines was also investigated. This is the first report on the chemical content of the hydrodistilled dried ground rhizome oil of* temu tis* and its antiproliferative activity against selected human cell lines.

## 2. Materials and Methods

### 2.1. Collection of Plant Materials and Extraction of Rhizome Oil

The dried rhizomes of* temu tis* were collected from Yogyakarta, Indonesia, in September 2012. A voucher specimen, KL 5793, was deposited at the herbarium of the Chemistry Department, Faculty of Science, University of Malaya, Kuala Lumpur, Malaysia. The rhizomes (300.00 g) were cut into small pieces, ground, and immediately soaked in distilled water (1.00 L) in a round-bottomed flask. The soaked sample was distilled in a Clevenger-type apparatus for 5 hours. The yield of the oil (2.16 g, 0.72%) was calculated based on the weight of the dried plant material.

### 2.2. GC-MS and GC-FID Analysis

The oil was analysed on an Agilent Technologies 7890A GC system equipped with an FID detector using a fused HP-5 silica capillary column (5% diphenyl- and 95% dimethyl-polysiloxane, 30.00 m × 0.32 mm ID, 0.25 *μ*m film thickness) with helium as the carrier gas at a flow rate of 1.0 mL per minute. The column temperature was initially programmed to and kept at 60.0°C for 10.0 minutes, then increased at 3.0°C per minute to 230.0°C, and held for 1.0 minute. The temperatures of the injector port and detector were set to 230.0°C and 250.0°C, respectively, with a split ratio of 1 : 20. GC-MS analysis was performed on an Agilent Technologies 6890N GC System equipped with a 5975 inert mass selective detector (70 eV direct inlet) on an HP-5ms capillary column (30.0 m × 0.25 mm ID, 0.25 *μ*m film thickness). The column temperature was programmed to 60.0°C for 10.0 minutes, then increased to 230.0°C at 3.0°C per minute, and held for 1.0 minute at 230.0°C. Helium was used as the carrier gas at a flow rate of 1.0 mL per minute with a split ratio of 1 : 20. The injector port temperature was set to 230.0°C and detector to 250.0°C. The obtained total ion chromatogram was autointegrated by ChemStation, and the components were identified by comparison with an accompanying mass spectral database [11]. The arithmetic index (AI) was experimentally measured from the programmed temperature GC-FID by arithmetic interpolation between bracketing alkanes, using a homologous series of n-alkanes as standards [12, 13].

### 2.3. Cell Propagation

Human breast carcinoma cells (MCF7), human cervical carcinoma cells (Ca Ski), human lung carcinoma cells (A549), human colon carcinoma cells (HCT116 and HT29), and noncancerous human lung fibroblast cells (MRC5) were purchased from the American Tissue Culture Collection (ATCC, USA). All the cells were cultured using protocols described earlier [14]. MCF7, Ca Ski, A549, and HT29 cells were cultured in supplemented RPMI 1640 medium, HCT116 cells in supplemented McCoy's 5A medium, and MRC5 in supplemented Eagle's minimum essential medium (EMEM). The RPMI 1640, McCoy's 5A, and EMEM media (Sigma-Aldrich, USA) were supplemented with 10.0% v/v foetal bovine serum (PAA Laboratories, Austria), 100.0 *μ*g/mL penicillin/streptomycin (PAA Laboratories, Austria), and 50.0 *μ*g/mL amphotericin B (PAA Laboratories, Austria). The EMEM medium was also supplemented with 11.0 mg/mL sodium pyruvate (Sigma-Aldrich, USA). Briefly, the cells were cultured in 25.0 cm^3^ tissue culture flasks (Nunc, Denmark) in 5.0% CO_2_ in an air-jacketed incubator (ESCO, USA) kept at 37.0°C in a humidified atmosphere and routinely observed under inverted microscope (Carl Zeiss Axio Vert.A1, Germany) for any contamination. The medium was replaced every 2 or 3 days until cell confluence was achieved, and the cells were detached using Accutase (PAA Laboratories, Austria). The rhizome oil of* temu tis* was dissolved in molecular-biology-grade dimethyl sulfoxide (DMSO) from Sigma-Aldrich, USA.

### 2.4. Antiproliferative Assay

The antiproliferative assay was performed using an MTT assay according to the method of [15] with some modifications. Cells were plated into sterile 96-well culture plates at a density of 3.0 × 10^4^ cells/mL. The 96-well plates were incubated in 5.0% CO_2_ in an air-jacketed incubator at 37.0°C for 24 hours for the cells to adhere. The medium was removed after 24 hours of incubation, and 150.0 *μ*L of fresh medium containing various concentrations of the rhizome oil of* temu tis* was added. The plates were further incubated for 72 hours. Then, 20.0 *μ*L of MTT solution (Sigma-Aldrich, USA) was added to each well, and the plates were again incubated for 4 hours. Medium containing MTT was discarded, and 150.0 *μ*L of DMSO was added into each well to dissolve the formazan crystals. The absorbance of each well was measured at 570 nm using a microplate reader (Synergy H1 Hybrid Multi-Mode Microplate Reader, USA). The IC_50_ values were determined by the interpolation of the dose-response curve for each cell line.

## 3. Results and Discussion

### 3.1. Yield of Rhizome Oil Extraction

**Figure 1:**
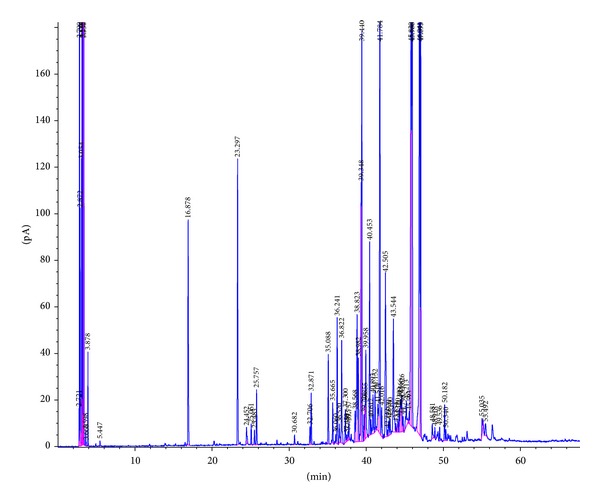
The GC-FID profile of the essential oil of rhizomes of* Curcuma purpurascens* Bl. (*temu tis*).

Hydrodistillation of the dried rhizomes of* temu tis* (300.0 g) yielded 2.2 g (0.7%) of oil. The identified constituents of the rhizome oil are listed in Table 1. The constituents were identified by matching their mass spectra and arithmetic indices with reference libraries [11–13]. Thirty-four (34) compounds consisting of eight (8) monoterpenoids (9.7%), fifteen (15) sesquiterpenes (22.2%), and eleven (11) sesquiterpenoids (56.2%) were identified from the rhizome oil of* temu tis*. The monoterpenoids are 1,8-cineole, camphor, borneol, terpinen-4-ol,* p*-cymen-8-ol, *α*-terpineol, thymol, and piperitone, while the sesquiterpenes are *δ*-elemene, *β*-elemene, cis-*α*-bergamotene, trans-caryophyllene, *γ*-elemene, aromadendrene, *α*-humulene, trans-*β*-farnesene, *γ*-muurolene,* ar*-curcumene, *α*-selinene, *β*-bisabolene, *β*-sesquiphellandrene, selina-3,7(11)-diene, and germacrene-B. The identified sesquiterpenoids are curzerene,* ar*-turmerol, guaiol, trans-*β*-elemenone, *γ*-eudesmol, atractylone,* ar*-turmerone, turmerone, germacrone, and curlone. These components made up 88.1% of the total components detected in the GC chromatogram of the oil (Figure 1). The major components identified were turmerone (13.5%), germacrone (13.2%),* ar*-turmerone (9.4%), germacrene-B (8.8%), and curlone (6.2%), which together amount to 51.1% of the total oil.

### 3.2. GC-MS and GC-FID Analysis

The findings of this study differ from a previous study on the rhizome oil of* temu tis*, which reported turmerol and* ar*-turmerone as the major components [8]. The current study also did not find xanthorrhizol in the rhizome oil of* temu tis*. This study identified thirty-four (34) compounds in the rhizome oil of* temu tis*, but the previous study identified only turmerone,* ar*-turmerone, xanthorrhizol, and curzerene [8]. Ground rhizomes of* temu tis* have been used as adulterants in ground common turmeric and* C. xanthorrhiza* (*temulawak*) for higher profit margins [8]; thus, it is necessary to compare the chemical content of its rhizome oil with those of common turmeric and* temulawak*. Previous studies on the essential oil content of dried rhizomes of common turmeric reported* ar*-turmerone as the major component, ranging from 21.4% to 49.0% [16–18], while the essential oil content of fresh rhizomes showed* ar*-turmerone content ranging from 24.4% to 49.1% [16, 19, 20]. Others reported *α*-turmerone as the major component ranging from 21.4% to 44.1% [21, 22].* Ar*-curcumene (40.0% to 65.0%) was reported to be the major compound identified from the rhizome oil of* temulawak* [23, 24], followed by xanthorrhizol (21.5%) [24]. A recent report identified xanthorrhizol as the major component (31.9% to 64.4%) in the rhizome oil of* temulawak* [25, 26]. Hence, the results of chemical investigations on the rhizome oil of* temu tis* in this study differ from those reported in the literature for both common turmeric and* temulawak*. The presence of* ar*-turmerone and xanthorrhizol found in the essential oil of* temu tis* by the Indonesian researchers in the previous study could possibly be due to adulteration by common turmeric and* temulawak* in the sample used [8].

The yield of the rhizome oil of* temu tis* reported in the previous study was 4.6% [8], while that of the rhizome oil of* temu tis* obtained in this study is only approximately 0.7%. A previous study on the rhizome oil of* temulawak* showed that the yield of the rhizome oil from* temulawak* is approximately 4.5% [25], while the yield of essential oil from both fresh and dried rhizomes of common turmeric ranged from 0.7% to 1.1% [17, 19, 21, 22]. The similarity of the yield of the rhizome oil of* temu tis* obtained by the Indonesian researchers (4.6%) with that obtained by Jantan et al. [25] again indicated that the ground* temu tis* used in their study could have been adulterated with* temulawak*.

### 3.3. Antiproliferative Effect

The antiproliferative effects of the rhizome oil of* temu tis* against human breast carcinoma cells (MCF7), human cervical carcinoma cells (Ca Ski), human lung carcinoma cells (A549), human colon carcinoma cells (HCT116 and HT29), and noncancerous human lung fibroblast cells (MRC5) were investigated via an MTT assay, and the results are shown in Table 2. According to the US NCI's plant screening program, crude extracts with IC_50_ values (concentration that inhibited 50% of cell proliferation) of less than 20.0 *μ*g/mL following incubation between 48 and 72 hours are considered to have* in vitro* cytotoxic activity [27]. The rhizome oil of* temu tis* exhibited very good cytotoxic effects against HT29 cells with an IC_50_ value of 4.9 ± 0.4 *μ*g/mL. However, it exhibited no inhibitory effect against MCF7 cells and very mild cytotoxicity against A549, Ca Ski, and HCT116 cells with IC_50_ values of 46.3 ± 0.7, 32.5 ± 1.1, and 35.0 ± 0.3 *μ*g/mL, respectively. The rhizome oil of* temu tis* also exhibited mild cytotoxicity against the noncancerous human lung fibroblast cell line, MRC5 (IC_50_ value of 25.2 ± 2.7 *μ*g/mL).

There are few reports found in the scientific literature on the antiproliferative effect of the rhizome oil of* Curcuma* species. Two studies reported that the rhizome oil of* Curcuma zedoaria* exhibited an inhibitory effect on the growth of a human promyelocytic leukaemia cell line (HL-60) and nonsmall cell lung carcinoma cell lines (H1299, A549, and H23) with IC_50_ values of 500.0, 80.0, 80.0, and 185.0 *μ*g/mL, respectively [28, 29]. The rhizome oil of* Curcuma wenyujin* had an antiproliferative effect on the human hepatoma cell line, HepG2, with an IC_50_ value of 70.0 *μ*g/mL [30]. However, the reported antiproliferative effects for the rhizome oil of both* Curcuma zedoaria* and* Curcuma wenyujin* did not meet the definition of* in vitro* cytotoxic activity defined by the US NCI's plant screening program [27] because their IC_50_ values are greater than 20.0 *μ*g/mL following 72 hours of incubation. Thus, the rhizome oil of* temu tis* exhibited better antiproliferative effects against the investigated human carcinoma cell lines in comparison to those reported in the literature [28–30], and the obtained IC_50_ value against HT29 cells is less than 20.0 *μ*g/mL following 72 hours of incubation.

The good cytotoxic effect exerted by the rhizome oil of* temu tis* against HT29 cells may be due to the presence of an appreciable amount of* ar*-turmerone (9.4%) and germacrene-B (8.8%) in the rhizome oil. Germacrene-B was reported to exhibit good cytotoxicity against a human ovarian cell line, A2780, with an IC_50_ value of 6.4 *μ*g/mL; however, presently, there is no report on the molecular pathway of cancer cell death induced by germacrene-B [31].* Ar*-turmerone was reported to have an inhibitory effect against several cell lines, namely, a human myelogenous leukaemia carcinoma cell line (K562), a human histiocytic lymphoma carcinoma cell line (U937), a mouse lymphocytic leukaemia carcinoma cell line (L1210), and a rat basophilic leukaemia carcinoma cell line (RBL-2H3), with IC_50_ values between 20.0 and 50.0 *μ*g/mL [32].* Ar*-turmerone was also reported to induce apoptosis in HepG2 cells (a human hepatocellular carcinoma cell line) via reactive oxygen species- (ROS-) mediated activation of extracellular signal-related (ERK) and c-Jun N-terminal (JNK) kinases, which then trigger both extrinsic and intrinsic caspase cascades, leading to apoptosis [33].

In this study, the rhizome oil of* temu tis* was found to exhibit a strong inhibitory effect against HT29 cells but very mild cytotoxicity against HCT116 cells, even though both cells are human colon carcinoma cell lines. HT29 cells express cyclooxygenase-2 (COX-2), while HCT116 cells do not [34]. The expression of COX-2 protein is elevated during colon carcinogenesis [35]. A recent study reported that* ar*-turmerone blocked the NF-*κ*B, PI3K/Akt, and ERK1/2 signalling pathways in human breast cancer cells, which led to suppression of 12-*O*-tetradecanoylphorbol-13-acetate- (TPA-) induced upregulation of matrix metalloproteinase- (MMP-) 9 and COX-2 expression [36].* Ar*-turmerone (9.4%) in the rhizome oil of* temu tis* may have suppressed COX-2 expression by blocking the NF-*κ*B, PI3K/Akt, and ERK1/2 signalling pathways, and synergistic effects of other constituents, such as turmerone, germacrone, germacrene-B, and curlone, may have inhibited the proliferation of HT29 cells. However, the amount of* ar*-turmerone was insufficient to exert any inhibition against the proliferation of MCF7 cells. Further* in vivo* toxicity studies on animal models will be attempted to determine the toxicity of the rhizome oil of* temu tis*, as it showed a mild inhibitory effect against MRC5 cells, the noncancerous human lung fibroblast cell line (IC_50_ value of 25.2 ± 2.7 *μ*g/mL).

## 4. Conclusion

In conclusion, GC-MS analysis of the rhizome oil of* temu tis* could be developed into a standard method to identify and differentiate the rhizome of* temu tis* from the rhizomes of common turmeric and* temulawak*. The rhizome oil of* temu tis* exhibited selective cytotoxic effect towards HT29 cell lines. Its inhibitory effect against HT29 cells may be due to the augmentation of COX-2 expression levels by* ar*-turmerone and synergistic effects of other constituents, such as turmerone, germacrone, germacrene-B, and curlone. In addition, the data obtained from this study provide a scientific basis for the use of this rhizome oil in the treatment of cancer-related diseases.

## Figures and Tables

**Table 1:** Chemical composition of the essential oil of *temu tis*' rhizomes.

Number	Compounds	Arithmetic indices (AI)		Percentage of peak area	Methods of identification
		Calculated	Literature [13]		
1	1,8-Cineole	1031	1026	3.3	MS, AI
2	Camphor	1144	1141	4.0	MS, AI
3	Borneol	1166	1165	0.3	MS, AI
4	Terpinen-4-ol	1178	1174	0.3	MS, AI
5	*p*-Cymen-8-ol	1184	1179	0.2	MS, AI
6	*α*-Terpineol	1191	1186	0.8	MS, AI
7	Thymol	1293	1289	0.1	MS, AI
8	*δ*-Elemene	1339	1335	0.2	MS, AI
9	Piperitenone	1342	1340	0.7	MS, AI
10	*β*-Elemene	1393	1389	1.2	MS, AI
11	cis-*α*-Bergamotene	1416	1411	0.1	MS, AI
12	trans-Caryophyllene	1421	1417	1.8	MS, AI
13	*γ*-Elemene	1435	1434	1.6	MS, AI
14	Aromadendrene	1441	1439	0.1	MS, AI
15	*α*-Humulene	1455	1452	0.1	MS, AI
16	trans-*β*-Farnesene	1458	1454	0.2	MS, AI
17	*γ*-Muurolene	1478	1478	0.5	MS, AI
18	*ar*-Curcumene	1484	1479	2.6	MS, AI
19	*α*-Selinene	1488	—	1.3	MS
20	Curzerene	1500	1499	5.8	MS, AI
21	*β*-Bisabolene	1510	1505	0.4	MS, AI
22	*β*-Sesquiphellandrene	1526	1521	2.7	MS, AI
23	Selina-3,7(11)-diene	1544	1545	0.6	MS, AI
24	Germacrene-B	1561	1559	8.8	MS, AI
25	*ar*-Turmerol	1580	1580	3.3	MS, AI
26	Guaiol	1595	1600	0.3	MS, AI
27	trans-*β*-Elemenone	1607	1602	2.5	MS, AI
28	*γ*-Eudesmol	1633	1630	0.7	MS, AI
29	*β*-Eudesmol	1637	—	0.7	MS
30	Atractylone	1654	1657	0.6	MS, AI
31	*ar*-Turmerone	1671	1668	9.4	MS, AI
32	Turmerone	1675	—	13.5	MS
33	Germacrone	1702	—	13.2	MS
34	Curlone	1705	—	6.2	MS

		Total rhizome oil =		88.1%	

Remarks: MS: mass spectroscopy [11]; AI: arithmetic indices [13].

**Table 2:** Cytotoxicity activity of the rhizome oil of *temu tis*.

	Cell lines					
	MCF7	Ca Ski	A549	HCT116	HT29	MRC5
IC_50_ values (*μ*g/mL)	>100.0	32.5 ± 1.1	46.3 ± 0.7	35.0 ± 0.3	**4.9 ± 0.4**	25.2 ± 2.7

Tabulated values are mean standard error (SE, SE < 5.00) of three replicates.
